# Motivations and concerns about adolescent tuberculosis vaccine trial participation in rural Uganda: a qualitative study

**DOI:** 10.11604/pamj.2015.22.76.7097

**Published:** 2015-09-30

**Authors:** Esther Buregyeya, Asli Kulane, Juliet Kiguli, Phillipa Musoke, Harriet Mayanja, Ellen Maeve Hanlon Mitchell

**Affiliations:** 1 Makerere University School of Public Health, Kampala, Uganda; 2 Institute of Global Health, Department of Public Health Sciences, Karolinska Institutet, Stockholm, Sweden; 3 Makerere University School of Medicine, Kampala, Uganda; 4 KNCV Tuberculosis Foundation. The Hague, Netherlands; 5 Department of Global Health, Academic Medical Center, Amsterdam Institute of Global Health and Development, Amsterdam, Netherlands

**Keywords:** Willingness to participate, hypothetical tuberculosis vaccine, trial, adolescents, qualitative study, Uganda

## Abstract

**Introduction:**

Research is being carried out to develop and test new potentially more effective tuberculosis vaccines. Among the vaccines being developed are those that target adolescents. This study explored the stakeholders’ perceptions about adolescent participation in a hypothetical tuberculosis vaccine trial in Ugandan adolescents.

**Methods:**

Focus group discussions with adolescents, parents of infants and adolescents, and key informant interviews with community leaders and traditional healers were conducted.

**Results:**

The majority of the respondents expressed potential willingness to allow their children participate in a tuberculosis vaccine trial. Main motivations for potential participation would be being able to learn about health-related issues. Hesitations included the notion that trial participation would distract the youths from their studies, fear of possible side effects of an investigational product, and potential for being sexually exploited by researchers. In addition, bad experiences from participation in previous research and doubts about the importance of research were mentioned. Suggested ways to motivate participation included: improved clarity on study purpose, risks, benefits and better scheduling of study procedures to minimize disruption to participants’ academic schedules.

**Conclusion:**

Findings from this study suggest that the community is open to potential participation of adolescents in a tuberculosis vaccine trial. However, there is a need to communicate more effectively with the community about the purpose of the trial and its effects, including safety data, in a low-literacy, readily understood format. This raises a challenge to researchers, who cannot know all the potential effects of a trial product before it is tested.

## Introduction

Globally, one out of every three people is estimated to be infected with the causative agent *Mycobacterium tuberculosis*[1]. The World Health Organization (WHO) aims to eradicate tuberculosis (TB) as a public health problem by 2050 [2]. However, eradication will be impossible without an effective vaccine [1]. Currently the only licensed TB vaccine, Bacille Calmette-Guerin (BCG) is given early in life and results in variable and waning immunity to TB [3]. Therefore, research is being carried out to develop and test new potentially more effective TB vaccines that could substitute or complement BCG [4–8]. Among the vaccines being developed are those that target infants and also adolescents [1]. Adolescents are also considered a target group for TB vaccines due to the fact that the rate of TB infection increases steeply in adolescence [9]. Since the TB incidence is highest among developing countries, it is these countries that will benefit the most from the development of a new TB vaccine [10]. Therefore, for scientific, ethical and economic reasons, phase III TB vaccine trials, should ideally take place in communities where the incidence of infection and/or disease is high [10]. Many populations in developing countries like Uganda do meet the criteria. Thus, many volunteers living in high-burden areas are needed for phase II and III efficacy TB vaccine trials. This raises one important principle of research ethics, i.e. informed consent. Informed consent is a process (occurs at enrolment and throughout the study), not just a form signed for the legal protection of an institution [11]. It is based on complete revelation of the facts about the study. It is meant to inform potential participants about the purpose, risks, potential benefits, alternatives to research and their rights as study subjects. This is to allow people to make intelligent decisions whether or not to participate based on their own goals and values [12].

While the above views are clear, the question is how eager are communities in high burden settings to allow their youth to participate in TB vaccine trials. What are their views regarding research in general and TB vaccine trials in particular? The preparedness of the local communities needs to be assessed. Therefore understanding willingness of parents to allow their children to participate in vaccine trials is critical. Very few studies have reported willingness to participate (WTP) in TB vaccine trials and the factors associated with this willingness [13, 14]. A study on WTP in TB vaccine trials among adolescents in South Africa, found that acceptability depended on the invasiveness of study procedures [13]. Among adults living with HIV, willingness to be vaccinated with a hypothetical effective and licensed TB vaccine was found to be high, at 84.5% and 92.6%, respectively [14]. The factors associated with willingness to be vaccinated included good TB knowledge and high perceived risk of contracting TB [14]. Majority of studies on WTP in vaccine trials concern HIV vaccines. In these studies the perceived benefits of the vaccine and altruism were the main factors associated with WTP in trials [15, 16]. In a study in Kenya on willingness of parents to have their children receive an infant HIV-1 vaccine, the majority (97%) were willing [17]. For those who were not willing, reasons for not wanting to enroll included concerns about side effects, partner objection, fear of discrimination, and HIV-1 acquisition [17]

Therefore, understanding the parents /care takers and potential participants’ willingness to take part in a trial is critical for planning potential TB vaccine trials. Uganda was one of the high TB burden countries which were identified as a potential venue to conduct new TB vaccine trials by EDCTP and Aeras. To our knowledge, no studies have examined parents/caretakers willingness to allow their adolescents participate in a TB vaccine trial in Uganda. Our study explored willingness of adolescents, parents and care takers in allowing their adolescents participate in future TB vaccine trials..

## Methods

The study was carried out in June and July 2008 in the Iganga/Mayuge Demographic Surveillance Site (DSS), located in eastern Uganda, about 120 km east of the capital Kampala. The DSS is predominantly rural (90% rural), but also partly semi-urban (Iganga Town). The main source of income in the area is subsistence farming (Iganga District). In 2008, the DSS population was 68,000 individuals in 12,000 households. The DSS was established in 2005 and the activities carried out include vital registration of births, deaths, pregnancies, and migration three times per year and have been described elsewhere [9]. In addition, this area has been well researched for special studies [18]. Therefore the community members were relatively familiar with health research. The DSS is found in the Busoga region, a traditional Bantu kingdom situated in south-eastern in present day Uganda. It is a cultural institution that promotes popular participation and unity among its people, through cultural and developmental programs for the improved livelihood. The Basoga, the people of Busoga, speak *Lusoga* and are the second largest ethnic group contributing about eight percent of the total population in Uganda [19]. The Busoga region is a patriarchal society and predominantly of the Islam religion. Main economic activity is farming and fishing [19]. HIV prevalence in this region is 5.5% [20].

### Study design

This was an explorative qualitative study using focus group discussions (FGDs) and Key informant interviews (KIs) in June- July 2008. The study was part of a larger study on capacity building to conduct phase III TB vaccine trials in the Iganga-Mayuge Demographic Surveillance Site (DSS), designed to estimate TB incidence among infants and adolescents. The current study's primary aim was to assess knowledge, attitudes and health seeking behavior towards TB in the communities [21] as well as community willingness to allow their children participate in a hypothetical TB vaccine trial. In this paper, we focus on the willingness to participate in TB vaccine trial aspects of the study.

### Data collection

FGDs and KIs were conducted. In this particular study, FGDs were used because they are naturally designed to elicit normative views and perceptions [22]. FGDs were held separately with male and female parents/caretakers of infant and adolescents, adolescent boys and girls. Twenty four FGDs were conducted, including six FGDs of young mothers/fathers/caretakers (aged <36 years) of children, six of mothers/fathers/caretakers of adolescents, six of mature mothers/fathers/caretakers aged (>36 years), two of adolescent girls in school, two for adolescent boys in school and two for adolescents out of school (one for girls and one for boys. KIs were conducted to get detailed information about community's views [23] with regard to necessary conditions and constraints on allowing their children to participate in a TB vaccine trial. Key informants included two local council leaders (LCs), two religious leaders (a Muslim and a Christian), two elders, two sub-county TB supervisors (health assistants) and two traditional healers. The KI respondents were chosen purposefully because they play a significant role in society and are opinion leaders upon who young parents look for advice. In addition, they were recruited because they are in constant touch with the community and therefore widely knowledgeable about community dynamics. FGDs and KIs were conducted at the village level. Individuals were identified with the help of the LCs. FGDS were grouped by such factors as age and sex, as homogeneity among focus group participants can facilitate sharing by eliminating status disparities. Study methodology has been described in detail elsewhere [21]. Topics for the interview guides, for both FGD and KI were designed to elicit participants’ views about WTP in a TB vaccine trial for the adolescents and willingness to allow their children participate, for the parents and caretakers.

### Data management and analysis

Data collection instruments for FGDs and KIs were pre-tested. Interviews with health workers were conducted in English, while the other interviews were conducted in the local language, Lusoga. Tape recordings and notes were used to record the interviews, which were later transcribed. All interviews were transcribed by moderators, and those in Lusoga were translated into English. Transcripts were read and re-read several times to get an overall picture. Analysis was carried out using content analysis method. Data were analyzed manually by coding related responses into themes [24]. These themes were shared among the authors for agreement.

### Ethical considerations

The study was approved by the Makerere University School of Public Health Institutional Review Board and the Uganda National Council for Science and Technology. The interviews were conducted only after informed consent was obtained from the study participants. Participation was entirely voluntary and the respondents were informed that at any time during the FGD or KI they could decide to opt out with no negative consequences. The interviews were carried out in confidential places.

## Results

Both individual interviews and FGDs revealed similar views; therefore, the presentation of results from the two groups is integrated. Where necessary, notes are made about the similarities or differences between the two sources of data. The data are presented according to the themes that emerged and illustrative quotes from respondents are used to exemplify the results.

### Willingness towards TB vaccine trial

Study participants expressed potential willingness to allow their children participate in a TB vaccine trial. Respondents’ most mentioned reason for participation was to be able to access learning opportunities about health related issues in their communities. *“When we get visitors like you (meaning researchers), we are able to get knowledge. We are able to learn how to remain healthy. So it's good to get involved.”* FGD young mothers. In one FGD for the elderly men, interest in participation in the hypothetical TB trial was altruistic, to help find a vaccine to protect their loved ones and the rest of the Ugandans. *“Because you have love for the life of people of Uganda”* FGD elderly men.

Though participants understood that the TB vaccine trial would be an experimental vaccine and were willing to have their children participate, there was a concern about the safety and side effects of taking part in unlicensed vaccine. Some KIs and FGDs mentioned they would be willing to allow their children participate, if assured that the trial was safe. *“Yes we will be willing, as long as it's not harmful.”* FGD mothers of adolescents

### Fears/concerns/inconveniences about new TB vaccine trial

#### Risk perception of the proposed vaccine trial

Respondents expressed their unwillingness to participate in a TB vaccine trial due the discomfort with the notion that they are “being experimented upon” and that the investigational product could be harmful. There was a feeling among some, that such activities like clinical trials (and even immunization with licensed vaccines) were deliberate strategies to control the population growth, particularly in Africa. A few FGDs and KIs also referred to an incident where children died following routine immunization. Community members wanted an assurance that the investigational vaccine would be safe for those who would receive it. Some preferred to await licensure. *“You people are accepting but some time back they vaccinated our children and there was an allegation that the ones who were immunized will not give birth”* FGD mothers of infants. *“Some of the parents may not accept. Some may think that it's a way of preventing their children from giving birth… Others think they want to reduce your life span. Laughter!”* FGD adolescent girls. *“Because of what happened (referring to the incident where children died following routine immunisation), I shall wait for others to take theirs, then I take mine.”* FGD mother infants. Elderly men were particularly suspicious of the placement of a trial in their community due to safety concerns about the vaccine. *“It should be tried in an area that's highly populated such that in case of anything, there is no effect on our population. Or why don't you try it on yourself (meaning researchers) first?”* FGD elderly fathers of infants. Two interviewees observed that if people really fully grasp the experimental nature of vaccine trials they will be scared and will not accept. *“You know people are very difficult, if you tell them it's a trial, they will not accept unless if you tell them that it has been tried elsewhere.”* Traditional healer. *“If you tell the people that it's a trial, they will have doubt and will be scared off.”* Head teacher

#### Side effects of the vaccine

Participants, even those who were willing to have their children participate, raised concerns mainly around potential safety issues. Most FGDs were concerned that the investigational product could have side effects on the study participants such as illness to their children, including TB or even death. *“Also parents need assurance that what they (the researchers) are going to do is good and won't harm their children.”* FGD young mothers of infants. *“The feared problem is that the adolescent may develop TB during the study and the parents will think that their child has got TB as a result of the new vaccine.”* FGD adolescent boys out of school

#### Discomfort caused by the trial procedures

Some of the FGDs and KIs were concerned about the potential discomfort associated with the study procedures such as injections in the event that a new vaccine candidate was to be administered by injections. *“One of the difficulties is the children might fear to be injected and so fails to turn up. If they hate taking tabs when sick, what will happen if they are to be injected?”* Women-Care takers adolescents

#### Inconveniences due to follow up

Following up of the participants was viewed as a good thing and would help the study team discover the side effects of the investigational product early. It was also interpreted as a sign that the study team cares about their study participants. Some viewed it as a privilege to have health workers visit them in their homes rather than them going to the health facility. Having a health worker visit them at home was seen as an opportunity to get answers to their health-related questions, saving them the transport to go to health facilities. However, some FGDs and KIs raised concerns about the follow up process, mentioning that it could lead to stigmatization of the participants and their families. An FGD participant in the young mothers group believed that being followed up might make people think that they were HIV infected. While in another FGD said that participants will be stigmatized and assumed to have TB. *“People would laugh at you thinking you have TB.”* FGD adolescent girls. *“This participant will not feel free with his other friends. They will keep on teasing him and telling him/her that he/she will get side effects of the vaccine.”* FGD adolescent boys out of school

#### Priority setting, who makes the decision and how?

In most KIs and FGDs, community members including the youths were perceived as not to have time due to many commitments. Participating in the proposed trial and other research activities was not viewed as a priority. This was commonly mentioned by FGDs from fathers. *“Some people could be willing but when they do not have time."Others say it's time wasting.”* FGD young fathers. It was also expressed that the study participants would lose valuable academic time due to the follow up process and this was commonly mentioned by the FGDs among adolescents. *“If it's exam time this (follow up) can disorganize you.”* FGD adolescent boys. *“The problem. I am sensing is if one is told to participate, say 3 times a week, it becomes time wasting. So one may decide to go and do his/her other duties since he/she will not be getting anything from the study.”* FGD adolescent boys out of school.

#### Benefits to the participants

The issue of how much youth benefit from participation in a vaccine trial and other research activities was raised.

#### Research ethics: Motives and behaviour of researchers

There was a perception that Ugandan and foreign researchers gain a lot out the research work compared to benefits accrued by the communities. This perception was common among men (KIs and FGDs), as exemplified by the following quotes from FGDs for men. *“Some people think that researchers benefit a lot yet they do not share that money with the information providers.”* FGD elderly men. *“People think it's the researchers who benefit. They come and ask you so many questions but you do not benefit in any way.”* FGD young fathers. It was also feared that the study team might try to take advantage of adolescents or family members, such as having sexual relationships with the study participants or their parents. *“One might lose her marriage because the man might think you have an affair with the researchers.”* FGD young mothers of infants.

#### Experience from previous research

Negative experiences in prior research participation within the DSS shaped expectations of TB vaccine trials. For example; lack of feedback and insufficient attention to community norms regarding sensitive issues was given as a reason for lack of interest in participating in the hypothetical new TB vaccine trial. In addition, some respondents mentioned that they were tired of research. *“Lack of feedback. Because many researchers come but they do not give us reports.”* FGD young men. *“At times experience from previous researchers who were not good.”* FGD Young fathers. Having a good relationship with the study participants was raised as an important means of keeping participants interested. One FGD for young women, it was mentioned that people are already fatigued with research and so the researchers were advised to pay more attention to the relationship with the communities to make their study appealing and thus be able to keep participants engaged. *“People are fed up of research, so you need to be very convincing.”* FGD mother of infants. *“It all depends on your communication skills. If you talk to people well, then they will be encouraged to continue participating.”* FGD mothers of adolescents

#### Illiteracy and lack of confidence

Another reason given for reticence was due to lack of formal education and being uninformed about the potential importance of research. Lack of confidence was mainly attributed to lack of formal education and this was felt to bring about difficulty in interacting with the researchers on an equal footing. Some FGDs reported that some people who lacked formal education felt inhibited by the use of English and fear that they would not grasp what the research is about. *“It depends on the level of understanding in the family. Some families are more enlightened and so want to learn more.”* FGD elderly men.

### Ways to motivate/encourage participation

#### Informed Consent

Though participants understood that this was a trial of an investigational product, sensitization to the parents and adolescents about the study objectives and the safety of the trial vaccine was deemed essential before they could make a decision. Most of the FGDs and KIs reported that the way to encourage them and their children participate in the study was by giving them comprehensive and readily understandable information about the investigational product. Participants felt that sensitizing the community about the purpose and procedures of the trial was critical on deciding whether to participate or not. Continuous sensitization and counselling about the importance of their participation was also reported to be necessary during the trial in order to encourage retention. Participants reported that even before deciding on whether to participate, they should be given information on how they will benefit from the trial. *“Tell us the purpose of this new vaccine, its name and people will be willing depending on how they would have understood.”* FGD young men *“In most cases people need to know the purpose of the study and how they will benefit before deciding to participate.”* FGD young men.

#### Whom to involve in decision making

In making a decision to participate in the proposed TB vaccine trial, all FGDs and KIs indicated that consulting their local leaders (LCs) was very important before deciding to participate in the investigational product trial. Information from LCs was reported to be the most trusted by the community members. Majority of the participants were of the view that researchers should go through their LCs when introducing the investigational product trial. In addition, the other important people to consult were their spouses, relatives, elders in the community and friends. Although there was concern about cooperation by village leadership expressed by at least one. *“After my wife, I can also involve my neighbour”* FGD elderly men. *“Any one older than me, local leaders, health workers in the area and anyone who has ever participated in research”* FGD participant young men. The gender and age hierarchies in the community were significant influences on decision making. Some women expressed that they did not have a decisive role in whether or not children would be allowed to participate. *“For us who are married, our husbands are the ones who make decisions and they may refuse the children to join the study; saying it"s time wasting.”* FGD mother of infants

#### Incentives to the participants

The majority of FGDs and KIs mentioned that the way to encourage participation was to give participants incentives as a way of motivating them and also compensating for their time spent in the trial. Suggestions varied from allowances, medical treatment and emotional solidarity in case of adverse events, mosquito nets, food, clothing and school fees. *“You need to compensate for our time. You find that we leave our work behind to come and participate in such things but in most cases its you people (the researchers) who benefit.”* FGD mother of infants. *“People expect immediate assistance in case they get in problems… Expectation to get some help in case you get problems, for example if you donate blood. This could help you in case you go to hospital”* FGD elderly men.

## Discussion

This study explored willingness of parents and care takers in allowing adolescents participate in future TB vaccine trials. Findings from this study suggest that community members may be willing to allow their children participate in a hypothetical TB vaccine trial under certain conditions. The reasons given for the interest to participate were the opportunity to learn about health related issues, to receive tangible benefits, and help their community. The decision to participate in research would hinge on the quality of information provided about the study, its purpose, risks and how they benefit (i.e. informed consent). Youth participation in research was not considered a priority by some, and was felt to detract them from academic and economic activities. Similarly, participants felt that to be encouraged to participate; they should be compensated for their time they would spend in the trial or be given incentives, since researchers also get paid. Bad experiences in previous research and the fatigue from research participation were reported as negative influences upon future trials. There was fear that the investigational product could have bad side effects and so an assurance that the investigational product is safe was critical before participation. The issue of assuring potential participants that a trial product is safe and without risks, is at odds with the clinical uncertainty that is at the heart of phase III clinical trials. This precondition represents a dilemma for trialists since a full safety profile is never available at the time of an efficacy trial. In vaccine development the side effects of the trial product are not fully known and so the researchers cannot disclose all the potential side effects of the trial product [11]. This calls for both an ethical guidance as well as a personal determination whether the balance between potential benefit and potential harm is a positive one. Researchers in vaccine trials have to be as honest as possible. Though this might inhibit participation, the researchers have to give the right information to ensure that the potential participants understand the nature of the study and why they are being asked to participate, with emphasis that risks are not yet known and also the benefits to the study participants. This process has to continue throughout the study [25]. The importance of providing information about the research to assist in decision making process to participate in research has been reported by Mahomed in S. Africa [13].

In addition, previously it was thought that populations in Africa may not grasp the many layers of informed consent [25]. The findings in this study show this is false. Respondents were demanding to be given details of the trial, i.e. its purpose, risks and the benefits involved before they could make a decision to participate. They went on to emphasise that to encourage continuous participation/retention in the study, this information about the study has to be continuously given, which is in line with the principle of informed consent [26]. Youth's fear of being teased because of participation in a TB vaccine trial was raised. This hesitation was similar to what was reported in a study by Sahay et al in India [27]. The issue of how participants personally benefit from the study was also mentioned. The benefits tended to be more of individual (materialistic) than to a broader society (altruism) [27, 28]. The community's motivation to participate in research including being treated well, receiving medical treatment, opportunity to learn, and incentives like food and allowances has been found in other studies [27–32]. However, it has been argued that some of these incentives, like improved medical care provided during the trial may constitute an undue inducement and hence be considered coercive [33]. Therefore, compensation of the trial participants should be fair, not to induce participation [33]. Respondents suggested that the researchers should test the investigational product on themselves first is similar to what was found elsewhere [27, 30] where some felt that they were singled out to be used as “guinea pigs” and being exploited because of their race [27, 30, 31]. This reveals the level of mistrust of researchers’ motives and rationales [31]. Decision making process was reported to involve consulting the local leaders, spouses, elders and relatives. This is similar to what had been reported elsewhere in Africa [25]. Previous studies in various African countries show that for better or for worse, community leaders are the gatekeepers of their communities [25, 34]. This underscores the significance of researchers going through the community leaders before seeking consent from the study participants, though this does not replace the consent from individuals. This study brings out the importance of continuous sensitization to the study participants before enrolment and throughout the study period as one of the factors to enhance participation. Provision of study information is related to trust [27, 31] and that continuous information and communication during the study is more effective in facilitating enrolment and retention than the information provided during the consent process alone [31]. This is in line with the principle of informed consent [26]. Participants need to be aware of the autonomy to leave the trial at any time. Like most qualitative study results, the findings of this study cannot be generalized beyond the context we studied. It explores WTP in a hypothetical TB vaccine trial among adolescents and of their parents/caretakers and therefore may not reflect the actual WTP and consent when a specific, concrete set of risks and benefits are presented. However, use of these qualitative methods facilitated richer understanding of the barriers and facilitators in allowing adolescents participate in a TB vaccine trial. In addition, this study had a wide community representation i.e. views from caretakers/parents, adolescents and opinion leaders. This representation may have given rich and representative views of the community at large.

## Conclusion

The findings from this study provide a valuable insight on the barriers of implementing vaccine trials in developing countries. Informed consent and how participants benefit were stressed as important things to be considered in planning for these trials. Researcher's behaviour and trustworthiness was also considered critical. Since this was a hypothetical study on WTP to participate in TB vaccine trial, true findings can be got by actual participation in a trial.
